# Autogenic splenic implantation versus splenectomy in patients undergoing distal pancreatectomy for benign or low-grade malignant lesions of the distal pancreas: study protocol for a multicentre, open-label, randomized controlled trial (RESTORE)

**DOI:** 10.1186/s13063-023-07714-1

**Published:** 2024-01-09

**Authors:** Mohammed Abu Hilal, Christoph Kuemmerli, Jasper P. Sijberden, Alma Moekotte, Giuseppe Zimmitti, Adnan Alseidi, Horacio J. Asbun, Ravi Marudanayagam, Morgan Bonds, Filipe Kunzler, Robert Sutcliffe, Efrem Eren, John N. Primrose, Anthony P. Williams

**Affiliations:** 1 Department of Surgery, Fondazione Poliambulanza Istituto Ospedaliero, Via Leonida Bissolati, 57, 25124 Brescia, Italy; 2 Department of Surgery, University Hospital Southampton NHS Foundation Trust, Tremona Road, Southampton, SO16 2YD UK; 3 Department of Surgery, Amsterdam UMC Location, University of Amsterdam, Amsterdam, The Netherlands; 4 Division of Hepatopancreatobiliary and Endocrine Surgery, Virginia Mason Medical Center, Seattle, WA USA; 5 Department of Surgery, University of California - San Francisco, San Francisco, CA USA; 6 Division of Hepatobiliary and Pancreas Surgery, Miami Cancer Institute, Miami, FL USA; 7 Department of Surgery, University Hospitals Birmingham NHS Foundation Trust, Birmingham, UK; 8 NIHR Southampton Clinical Research Facility, NIHR Southampton Biomedical Research Centre and Southampton NIHR CRUK Experimental Cancer Medicine Centre, University Hospital Southampton NHS Foundation Trust, Southampton, UK

**Keywords:** Splenectomy, Distal pancreatectomy, Overwhelming post-splenectomy infection syndrome, Autogenic splenic implantation

## Abstract

**Background:**

The spleen plays a significant role in the clearance of circulating microorganisms. Sequelae of splenectomy, especially immunodeficiency, can have a deleterious effect on a patient’s health and even lead to death. Hence, splenectomy should be avoided and spleen preservation during elective surgery has become a treatment goal. However, this cannot be achieved in every patient due to intraoperative technical difficulties or oncological reasons. Autogenic splenic implantation (ASI) is currently the only possible way to preserve splenic function when a splenectomy is necessary. Experience largely stems from trauma patients with a splenic rupture. Splenic immune function can be measured by the body’s clearing capacity of encapsulated bacteria. The aim of this study is to assess the splenic immune function after ASI was performed during minimally invasive (laparoscopic or robotic) distal pancreatectomy with splenectomy.

**Methods:**

This is the protocol for a multicentre, randomized, open-labelled trial. Thirty participants with benign or low-grade malignant lesions of the distal pancreas requiring minimally invasive distal pancreatectomy and splenectomy will be allocated to either additional intraoperative ASI (intervention) or no further intervention (control). An additional 15 patients who will undergo spleen-preserving distal pancreatectomy serve as the control group with normal splenic function. Six months postoperatively, after assumed restoration of splenic function, patients will be given a *Salmonella typhi* (Typhim Vi™) vaccine. The *Salmonella typhi* vaccine is a polysaccharide vaccine. The specific antibody titres immediately before and 4 to 6 weeks after vaccination will be measured. The ratio between pre- and post-vaccination antibody count is the primary outcome measure and secondary outcome measures include intraoperative details, length of hospital stay, 30-day mortality and morbidity.

**Discussion:**

This study will investigate the splenic immune function of patients who undergo ASI during minimally invasive distal pancreatectomy with splenectomy. The splenic immune function will be measured using the surrogate outcome of specific antibody titre after vaccination with a *Salmonella typhi* vaccine. The results will reveal details about splenic function after ASI and guide further treatment options for patients when a splenectomy cannot be avoided. It might eventually lead to a new standard of care making sometimes more demanding and time-consuming spleen-preserving procedures redundant.

**Trial registration:**

International Standard Randomized Controlled Trials Number (ISRCTN) ISRCTN10171587. Prospectively registered on 18 February 2019.

**Supplementary Information:**

The online version contains supplementary material available at 10.1186/s13063-023-07714-1.

## Background

The spleen initiates immune responses to blood-borne antigens, produces antibodies, and clears antibody-mediated pathogens. Some bacteria, in particular encapsulated bacteria, require opsonization to facilitate phagocytosis. In this case, memory B cells located in the spleen produce immunoglobulin M (IgM) that acts as an opsonin to enable the clearance of the polysaccharide-encapsulated bacteria like *Streptococcus pneumoniae*, *Neisseria meningitides*, *Haemophilus influenza* and *Streptococcus pyogenes* [1, 2]. Therefore, splenectomised individuals, with less memory B cells, are at lifelong risk of developing severe septic complications, which can potentially result in “overwhelming post-splenectomy infection (OPSI) syndrome” [3, 4]. While OPSI is relatively rare, with an incidence rate amongst splenectomised individuals of 0.13 per 100 person-years, it can rapidly progress from a mild flu-like illness to a fulminant sepsis with high mortality [4–6]. Due to this increased risk, splenectomised patients require regular vaccinations and, depending on national guidelines, a temporary or permanent commitment to prophylactic antibiotics [7].

Another role of the spleen is hemofiltration, maintaining the morphology and function of red blood cells by removing senescent and altered erythrocytes and particles such as Howell–Jolly bodies (HJB) [8–10]. In fact, a plethora of vascular complications have been described after splenectomy. They mostly arise due to an obstruction of blood vessels as a result of thrombocyte activation, hypercoagulability and a longer life span of erythrocytes and are more pronounced amongst patients with underlying haematological disease [11]. Furthermore, in 985 patients who underwent a splenectomy in conjunction with surgery for non-traumatic or non-malignant conditions of adjacent organs, a 40% increased risk of cancer was found 5–9 years later, with a significantly increased incidence of lung and ovarian cancer [12]. In addition, higher risks of head and neck cancer, digestive tract cancer and haematological malignancies have been reported [13, 14]. In contrast, other studies showed a lower incidence of cancer after splenectomy [15]. In fact, many cancers have been correlated with immunodeficiency, and the spleen plays an obvious role in the immune response. However, these processes are not yet entirely understood. To summarize, the spleen plays a relevant role in the body and, although barely understood, it is clear that splenic preservation is preferred.

Distal pancreatectomy (DP) often comes with concomitant splenectomy (DPS), as a result of the spleen having anatomical proximity to and sharing principal vessels with the body and tail of the pancreas. Furthermore, in the case of a malignancy, splenectomy is routinely performed for oncological reasons. For benign or low-grade malignant lesions such as mucinous cystic neoplasm (MCN), intraductal papillary mucinous neoplasm (IPMN), pseudocyst or chronic pancreatitis, splenectomy has no clear advantages and is associated with greater morbidity [16].

Interestingly, studies comparing the risk of severe post-splenectomy infection showed that rates of infection were lowest amongst patients who underwent a splenectomy because of splenic rupture [5, 17, 18]. This could be explained by spontaneous splenosis, which has been shown to occur in up to 65% of patients who underwent splenectomy for trauma. The phenomenon of spontaneous splenosis is presumed to be caused by seeding of splenic tissue after rupture of the capsule as a consequence of the trauma [19].

With this background, spleen preservation has become the goal in DP for benign conditions and neoplasms with low malignant potential [20]. However, if splenectomy is inevitable, autogenic splenic implantation (ASI) is a potential way to restore splenic function. During this procedure, pieces of splenic parenchyma are placed in the abdominal cavity, mostly in a surgically created pouch of the greater omentum. This results in regeneration of the splenic tissue, most likely as a result of the rich blood supply of the omentum [21, 22]. Multiple series of ASI have been described in the literature, the majority performed after splenectomy in trauma patients. These series have demonstrated that this procedure is safe and feasible with very few complications being reported [23].

Nevertheless, it remains unclear if ASI restores the splenic function, mainly due to difficulties to accurately test the splenic function. Spleen scintigraphy using technetium 99-m labelled erythrocytes or sulphur colloid verifies the presence, volume and perfusion of splenic tissue [24]. An actual qualitative statement about splenic function, however, is not possible. Assessment of blood films for the presence of HJB or pitted red blood cells is another measure reflecting phagocytic capacity. Nevertheless, the relation of the presence of HJB and splenic function remains unclear and morphological assessment is not sensitive [25, 26]. Lastly, experimental animal studies have suggested ASI might restore defects in the immune system of splenectomised individuals [22, 27, 28]. Therefore, the clearance of polysaccharide-encapsulated bacteria, the clinically most relevant function, has been used to assess immune function in humans. One way to measure clearance is by measuring specific antibodies against encapsulated bacteria. These enable opsonisation of bacterial capsules so that immune cells can recognize and attack them.

*Salmonella typhi* is an encapsulated bacterium and vaccination with the Typhim Vi™ polysaccharide vaccine can induce an antibody response in a controlled setting. Recently, a *Salmonella typhi* Vi IgG ELISA that can measure the antibody response has become available. So far, ASI has not been performed for the indication of distal pancreatectomy in an elective setting, whereas, this population might benefit even more as they are less likely to develop spontaneous splenosis due to posttraumatic seeding. In the present study, we aim to assess the splenic immune function after ASI performed during minimally invasive (laparoscopic or robotic) DPS as compared to minimally invasive DPS without ASI.

## Methods

### Study design

The study is an international multicentre, open-label, randomized controlled trial with parallel groups. Patients are randomized to the intervention (ASI) or control group (splenectomy) at two possible time points: prior to or during surgery. The patients in the control arm are presumed to have no splenic function. In addition, a third group, namely minimally invasive spleen-preserving distal pancreatectomy (SPDP), serves as a control with the assumed normal function of the spleen. The participants will be followed for 7 months and the study will be conducted over a 2-year period. Patients are recruited in six study sites with considerable expertise and a high annual volume in hepato-pancreatico-biliary surgery: The University Hospital Southampton; University Hospitals Birmingham; Morriston Hospital, Swansea (all in the UK); Fondazione Poliambulanza Istituto Ospedaliero in Brescia, Italy; Virginia Mason Medical Center, Seattle; and the Miami Cancer Institute, Miami (both USA).

### Participants

**Fig. 1:**
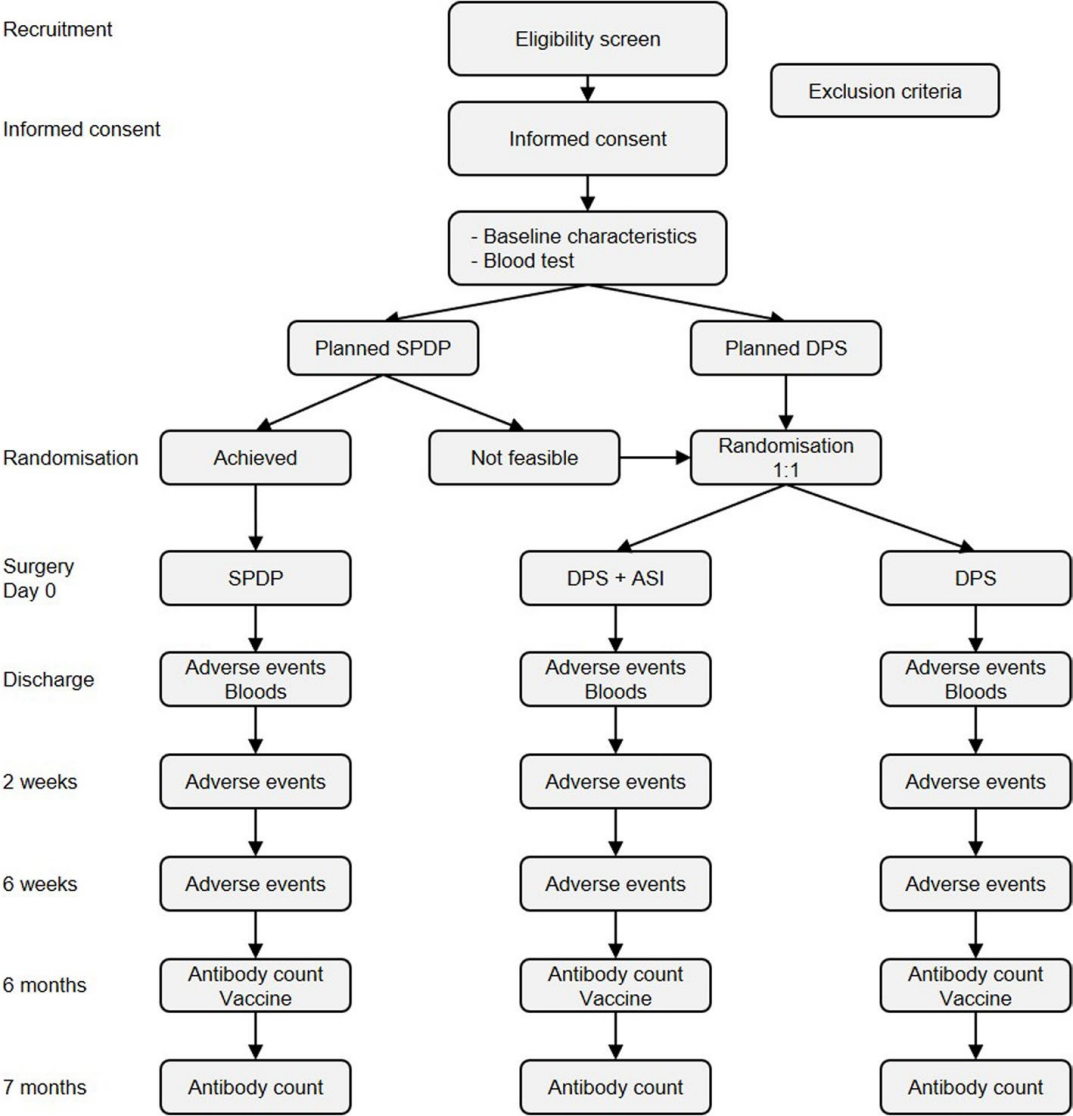
Trial flowchart. ASI, autogenic splenic implantation; DPS, distal pancreatectomy with splenectomy; SPDP, spleen-preserving distal pancreatectomy

All patients undergoing pancreatic surgery are discussed in a multi-disciplinary team (MDT) meeting. Prior to the MDT, a recent abdominal CT scan, an MRI and/or endoscopic ultrasound (EUS) are executed for the diagnosis and to determine the surgical approach (either minimally invasive DPS or SPDP) (Fig. 1).

**Fig. 2:**
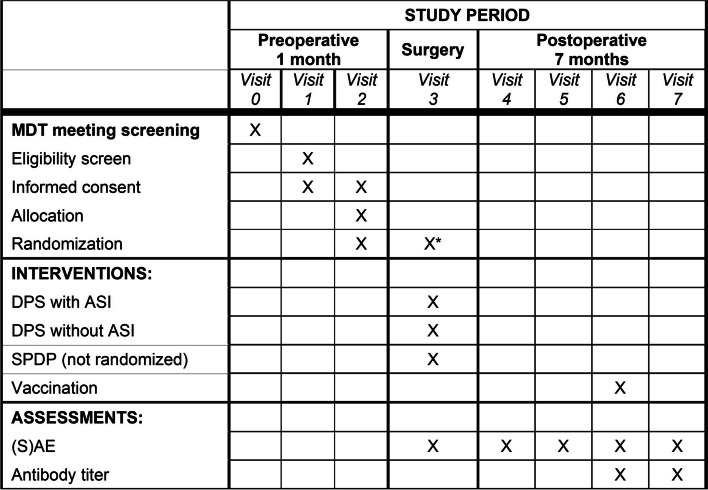
Schedule of enrolment, intervention and assessments. *If SPDP cannot be achieved, patients will be randomized to DPS with or without ASI

The inclusion criteria for this study are as follows: (1) patients are scheduled for elective minimally invasive DP with or without spleen preservation for suspected benign or low-grade malignant lesions (including low-grade neuroendocrine tumours) of the pancreatic body or tail; (2) patients must be fit to undergo distal pancreatectomy (American Society of Anaesthesiologists (ASA) classification ≤ 3) and (3) patients are at least 18 years old. Exclusion criteria comprise (1) proven or suspected pancreatic ductal adenocarcinoma; (2) asplenia (either functional, surgical or congenital); (3) any other known immune deficiency disorder; (4) known allergy to any of the Typhim Vi™ polysaccharide vaccine containing components; (5) pregnancy; and (6) lacking capacity to consent. If any of the exclusion criteria apply, the patients will be deemed not eligible for participation. Potential participants, who meet inclusion criteria, are approached by their treating surgeon during their outpatient visit. If the potential participant is interested in participating, a local researcher will further explain the trial in depth. Participants are offered at least 24 h to decide and consider informed consent (Fig. 2).

Routine preoperative blood samples are obtained for baseline analysis and fitness to undergo laparoscopic DP is assessed by an anaesthesiologist. In the case of a planned minimally invasive DPS, all patients will receive pneumococcal vaccination, *Haemophilus influenzae *type b conjugate vaccine and meningococcal conjugate vaccine according to national guidelines prior to or after surgery [7].

### Sample size calculation

A sample size calculation was performed using the expected outcomes of the primary endpoint based on the previous literature on the topic. Sánchez-Ramón et al. described that a cut-off point of threefold increase in antibody count following Typhim Vi™ polysaccharide vaccine results in a response rate of 99.9% in healthy persons and 40.3% in patients with common variable immunodeficiency [29]. Using these response percentages as estimator for the ASI and the DPS group responses respectively, a sample size of 12 in each arm will have a power of 80% to detect this difference at the 5% significance level using a *χ*^2^ square test. To account for lost to follow-up and dropouts, we aim to randomize 15 patients to each group. nQuery advisor v7.0 was used for the calculation. During accrual, the trial steering committee decided that the sample size needed to be increased to 55 patients due to a high dropout rate in the SPDP arm.

### Randomization and blinding

There are two possible moments of randomization: preoperative or intraoperative. If minimally invasive DPS is planned, patients will be randomized the day before admission to either ASI (intervention) or splenectomy (control). ASI will be performed at the end of procedure. If minimally invasive SPDP is the intended approach, splenectomy will only be performed if splenic preservation cannot be achieved due to technical or safety reasons during the procedure. In such a case, intraoperative randomization for ASI or splenectomy will be carried out on the spot, by a local researcher.

This will result in three groups:

1. Minimally invasive DPS with ASI (intervention)
2. Minimally invasive DPS without ASI (control)
3. Minimally invasive SPDP (control)

An online computer variable block randomization module between ASI and DPS in a 1:1 ratio will be used. Patients will receive a subject inclusion number and subject randomization number.

The current study is an open-label trial due to a variety of reasons. First, surgeons cannot be blinded when the study intervention precludes a surgical procedure. Second, the splenic function is not believed to be associated with specific “advantages” in the general population in Western countries. Hence, the patient may not have expectations of a better postoperative recovery with a functional spleen. Third, antibody count is an objective measurement that cannot be influenced by physicians, surgeons or patient’s attitude. As the design of the study is open label, with only outcome assessors being blinded, unblinding will not occur.

### Surgical technique

All patients will be allocated to undergo a minimally invasive DP. In case of predetermined SPDP, preservation of the spleen will always be attempted. In case of minimally invasive DP with splenectomy and ASI, minimally invasive DP with splenectomy will be performed according to the surgeon’s preference. At the end of the procedure, after retrieval of the specimen, ASI will be performed. The specimen is placed on a sterile field and about a third of the spleen, including capsule, is taken from the spleen. This part is cut into smaller pieces with a minimum total weight of 30 g [30]. These smaller pieces are weighted using a scale within a sterile field. As soon as 30 g of splenic tissue is reached, the fragments are placed into a newly created pouch of the greater omentum. Hemolock clips are used to create the pouch as well as for closure of the pouch after the splenic tissue is placed into the pouch. No vascular reconstruction is required. Postoperative care is based on enhanced recovery principles. Patients who undergo DPS, independent of ASI, will receive the standard of care according to national post-splenectomy guidelines that include lifelong prophylactic antibiotics and yearly influenza immunization [7].

### Primary outcome

The primary outcome is the clearance of polysaccharide encapsulated bacteria measured by titres of *Salmonella typhi*-specific antibodies just prior to vaccination 6 months after surgery and 4 to 6 weeks thereafter.

One 6-mL serum tube will be used for sample collection immediately prior to vaccination with Typhim Vi™. Pre-vaccination-specific antibody levels will be determined. The vaccine (Typhim Vi™, Sanofi Pasteur MSD) will be given immediately after taking baseline blood. The procedure for the second measurement of *Salmonella typhi*-specific antibody response is identical to the first one. Pre- and post-immunization serum samples are separated by centrifugation and subsequently stored in aliquots at ≤  − 20 °C until analysis. All samples will be shipped to the University Hospital Southampton and simultaneous performance of specific antibody testing will be carried out in the University Hospital Southampton after the last patient completes the 7-month follow-up period.

*Salmonella typhi*-specific antibodies are measured using a commercially available ELISA kit (VaccZyme™ Anti-S. typhi Vi human IgG EIA from The Binding Site Group Ltd., Birmingham, UK). Samples are run in duplicate following manufacturer instructions. The results of specific antibody levels to Typhim Vi™ are expressed as U/mL (normal range, 7.4–600 U/mL). The value of the response is given as the ratio between pre- and post-immunization antibody levels. A threefold increase between titres pre- and post-vaccination will be used to define a normal antibody response according to prior literature [31, 32]. Participants are advised not to have any other vaccinations during the study period.

### Secondary outcome

Intraoperative details (including operation time, estimated blood loss, blood transfusion), length of hospital stay, readmission rate, morbidity and mortality are the secondary outcomes. All complications are scored using the Clavien-Dindo classification of surgical complications [33].

### Data collection and management

Patient’s details will be de-identified and coded, and randomization will be performed in Castor (Castor EDC, Amsterdam, The Netherlands). Electronic data will be stored in eCRFs (Castor EDC, Europe-based server) and only research team members can access the CRF according to their rights given. Informed consent forms are collected in the study folder. Variables will be collected during study visits except the antibody count after 6 and 7 months, respectively, as these samples are processed altogether after all patients have completed the 7-month study visit. Only the principal investigator and the study coordinator will have access to the final data set. During the study period, patients are strictly followed up by the treating centres. All data produced by this study are considered confidential and will be handled according to the Data Protection Act 2018. All essential documents including source documents will be retained for a minimum period of 5 years following the end of the study by the study sites.

During the follow-up visits, participants were asked whether they were taking other medication that has an impact on the immune system. Adherence to the procedure to implant splenic tissue was ensured by an educational video and precise written instructions. Adherence was assessed by providing details about the number, size and weight of the implanted splenic fragments.

To improve participant retention, follow-up visits were scheduled prior to discharge, and for the 6-month follow-up, a range of ± 2 weeks was allowed. Patients were actively approached prior to the follow-up visits to confirm the date and time of the visit.

### Statistical analysis

For comparison of normally distributed continuous variables, the independent samples t-test will be used and values will be expressed as means and standard deviation. Continuous non-normally distributed variables will be compared using the Mann–Whitney *U* test and values will be expressed as medians (interquartile ranges). Categorical variables will be compared by chi-square or Fisher’s exact test as appropriate, and values will be expressed as proportions. A two-tailed *p*-value < 0.05 will be considered statistically significant. Risk ratios with 95% confidence intervals will be reported. Analysis of the outcomes will be performed based on the intention-to-treat (ITT) population. All randomized participants with an available antibody count will be included in the final analysis in the group they were assigned to. Missing data will not be imputed. The data from patients that do not complete the study will also be analysed. No additional subgroup analyses are planned. All data will be analysed using SPSS (IBM SPSS Statistics for Windows, version 26.0, Armonk, NY, USA).

### Oversight and monitoring

The trial steering committee from the coordinating centre consists of the principal investigator, a senior researcher from the University of Southampton and the trial coordinator. It oversees screening and patient enrolment, progression of the trial and monitors subject drop-out. During the set-up of the trial, the group met weekly and during the initial enrolment phase every 2 to 4 weeks. Monitoring visitations will be scheduled at an appropriate frequency. These visits will be conducted to evaluate the progress of the study, to ensure that the rights and well-being of the subjects are protected, to check that the reported clinical data are accurate, complete and verifiable from source documents, and if the conduct of the study is in compliance with the approved protocol with amendments and Good Clinical Practice. An independent data safety monitoring board (DSMB) will be formed and in the event of serious adverse events (SAEs) which may be related to the study, the DSMB will meet to review the data and advise the steering committee. The DSMB consists of the clinical experts and one senior statistician. Due to the small sample size and the planned analysis of the blood samples after the last follow-up, no interim analysis is planned. The trial will be stopped prematurely if there are serious safety concerns that might be related to any study-specific procedure, especially omental abscess formation within 30 days after the operation and consequent sequelae.

Random audits of trials by the sponsor by an independent auditing committee are allowed.

### Ethics

The conduct of the study will conform to the principles of the Declaration of Helsinki and relevant ethical guidelines. Ethical approval has been obtained from the South-Central Hampshire Research Ethics Committee (reference number 18/SC/0364). All substantial and non-substantial amendments will be submitted to an integrated research application system for approval by all relevant offices. Prior to any study-specific procedure, informed consent will be sought from all patients. On the consent form, participants are asked if they agree to use of their data should they choose to withdraw from the trial. Participants will also be asked for permission for the research team to share relevant data with people from the universities taking part in the research or from regulatory authorities, where relevant. Within this trial, no biological specimens are collected for storage.

There is no anticipated harm and compensation for trial participation. Post-trial care takes place according to centre-specific surveillance protocols.

The study data and statistical code will be available from the corresponding author on reasonable request, as is the full protocol. Results will be disseminated to participants, researchers and healthcare professionals through presentations at international and local conferences and online. Publication in a peer-reviewed scientific journal is planned with authorships for researchers of participating centres. The researchers declare no conflict of interest.

### Patient and public involvement

During the review of the initial protocol, patient representatives appointed by the funder provided input. The patient information sheet and informed consent form were reviewed by public and patient representatives and the research design was discussed. It was emphasized it would be most important to combine the research visits with regular follow-up visits to reduce the burden of study-related procedures.

## Discussion

Sequelae of splenectomy can be devastating and lethal [3, 4]. Whenever possible, splenic preservation after trauma or elective abdominal surgery should be attempted [20]. If splenic preservation cannot be achieved, the only potential way to preserve splenic function is ASI. Several centres have published case series of ASI in trauma patients, showing their positive experience in terms of safety and feasibility [23, 34]. However, splenic function measurements have not been carried out extensively and there is a lack of standardized reporting on how to measure the function of the spleen [26, 35, 36]. Mimicking an infection caused by encapsulated bacteria through vaccination, followed by measuring the specific antibody response, seems the most realistic surrogate outcome to assess splenic immune function. In this randomized trial, we assess splenic immune function using immunoglobulin count in response to a *Salmonella typhi* vaccination. The Typhim Vi™ polysaccharide vaccine was chosen over the non-conjugated Pneumovax 23® because of cross-reacting and different immunogenicity towards different serotypes and high pneumococcal pre-immunization levels in the population have been described, Whereas, the level of endemic infection with *Salmonella typhi*is lower [37, 38]. The newly developed *Salmonella typhi*Vi IgG ELISA measures the response to the Typhim Vi™ polysaccharide vaccine and allows quantifying the specific production of antibodies in response to the vaccination with higher power for discrimination than the Pneumovax 23® [29].

The decision to include only patients who are allocated to undergo minimally invasive surgery was made based on the following rationale: every surgery is an insult to the human body and, therefore, generates an inflammatory response. It has been suggested by several studies that the minimally invasive approach initiates less of an inflammatory response as compared to the open approach [39–42]. Hence, we felt that including only patients who will have minimally invasive surgery creates a more homogeneous study population and a fairer comparison.

There are some potential limitations to this study. First, we use only one measurement to assess splenic function. Nevertheless, we believe that the evaluation of the clearing function is clinically most relevant. Similarly, we intended to reduce additional radiation exposure to a minimum and eschew scintigraphy. As used in prior studies, post-ASI scintigraphy, in our opinion does not adequately reflect splenic function but rather only provides information that splenic tissue is present. Secondly, antibody titres will be assessed at the end of the study; therefore, interim analysis will not be performed, and futility of our interventions will be recognized only at the end of the study. However, clear stopping guidelines were based on the occurrence of postoperative SAEs. Finally, blinding of surgeons, patients and ward staff was deemed impossible due to the nature of the study.

To conclude, the results of this study may give details about splenic immune function after ASI and guide further treatment options for patients where splenectomy cannot be avoided. If splenic immune function can be preserved and the procedure is safe in an elective setting, ASI could become an alternative to the more demanding and time-consuming SPDP. Finally, if the immunologic function of the spleen is restored with ASI, prophylactic antibiotics for splenectomised patients may become obsolete resulting in huge cost saving.

## Trial status

Participant recruiting started on October 18, 2018; we expect to recruit the full sample until the end of May 2023. The 7-month follow-up visit of the last participants will be completed in November 2023. Protocol version 4.1; protocol version date 29 March 2022.

### Supplementary Information

**Additional file 1.**

## Data Availability

Not applicable.

## Abbreviations

ASI: Autogenic splenic implant
DP: Distal pancreatectomy
DPS: Distal pancreatectomy with splenectomy
HJB: Howell–Jolly bodies
MDT: Multi-disciplinary team
OPSI: Overwhelming post-splenectomy infection
(S)AE: (Serious) adverse events
SPDP: Spleen-preserving distal pancreatectomy
